# *Moraxella catarrhalis*: A Cause of Concern with Emerging Resistance and Presence of BRO Beta-Lactamase Gene—Report from a Tertiary Care Hospital in South India

**DOI:** 10.1155/2020/7316257

**Published:** 2020-02-07

**Authors:** Savitha Raveendran, Gauri Kumar, R. N. Sivanandan, Mary Dias

**Affiliations:** ^1^Department of Microbiology, St. John's Medical College Hospital, Bengaluru, India; ^2^Infectious Diseases Unit, St. John's Research Institute, Bengaluru, India

## Abstract

**Background:**

Found as a commensal in the upper respiratory tract, Gram-negative diplococcus *Moraxella catarrhalis* did not hold much importance as an infectious agent for long. The emergence of the first antibiotic-resistant strain of *M. catarrhalis* was noted in 1977 in Sweden. This has gradually spread worldwide over the years to more than 95% of the strains showing resistance to penicillin now. Penicillin resistance is mediated by the production of beta-lactamases encoded by bro-1 and bro-2 genes that code for beta-lactamases BRO-1 and BRO-2, respectively. The purpose of this study was to explore the trends of antibiotic resistance, the presence of bro genes, and clinical correlation of these findings with the rise in *M. catarrhalis* was noted in 1977 in Sweden. This has gradually spread worldwide over the years to more than 95% of the strains showing resistance to penicillin now. Penicillin resistance is mediated by the production of beta-lactamases encoded by bro-1 and bro-2 genes that code for beta-lactamases BRO-1 and BRO-2, respectively. The purpose of this study was to explore the trends of antibiotic resistance, the presence of bro genes, and clinical correlation of these findings with the rise in

**Methods:**

Strains of *M. catarrhalis* was noted in 1977 in Sweden. This has gradually spread worldwide over the years to more than 95% of the strains showing resistance to penicillin now. Penicillin resistance is mediated by the production of beta-lactamases encoded by bro-1 and bro-2 genes that code for beta-lactamases BRO-1 and BRO-2, respectively. The purpose of this study was to explore the trends of antibiotic resistance, the presence of bro genes, and clinical correlation of these findings with the rise in

**Results:**

Fourteen strains of *M. catarrhalis* was noted in 1977 in Sweden. This has gradually spread worldwide over the years to more than 95% of the strains showing resistance to penicillin now. Penicillin resistance is mediated by the production of beta-lactamases encoded by bro-1 and bro-2 genes that code for beta-lactamases BRO-1 and BRO-2, respectively. The purpose of this study was to explore the trends of antibiotic resistance, the presence of bro genes, and clinical correlation of these findings with the rise in

**Conclusion:**

The increase in antibiotic resistance and beta-lactamase production in *M. catarrhalis* is a cause of concern. The emerging resistance pattern emphasises the need for an appropriate antibiotic stewardship program in clinical practice. Importance should be given to the monitoring of the trends of antibiotic susceptibility and their usage to prevent the emergence of outbreaks with resistant strains and treatment failures.*M. catarrhalis* was noted in 1977 in Sweden. This has gradually spread worldwide over the years to more than 95% of the strains showing resistance to penicillin now. Penicillin resistance is mediated by the production of beta-lactamases encoded by bro-1 and bro-2 genes that code for beta-lactamases BRO-1 and BRO-2, respectively. The purpose of this study was to explore the trends of antibiotic resistance, the presence of bro genes, and clinical correlation of these findings with the rise in

## 1. Introduction

WHO has recognised antibiotic resistance as a global threat. What were earlier considered as harmless infections have now become very difficult to treat because they are caused by organisms that have developed resistance to commonly used antibiotics. One such organism, in which the rise in antibiotic resistance has been alarmingly high, is *Moraxella catarrhalis. M. catarrhalis* is a Gram-negative, aerobic, oxidase-positive diplococcus frequently seen as a coloniser of the upper respiratory tract [1].

The organism, in its pathogenic state, causes upper respiratory tract infections such as otitis media in children and lower respiratory tract infection in adults [1]. The severity of infection usually depends on the host's immune status.

After the identification of beta-lactamase-producing *M. catarrhalis* strains in 1977 in Sweden, there has been an unprecedented increase in the beta-lactam antibiotic resistance in this organism throughout the world [2]. More than 95% of global clinical isolates are now resistant to penicillin [3–6], and resistance to other classes of antibiotics is also on the rise. This might have led *M. catarrhalis* to become a well-established pathogen rather than an emerging one.

Beta-lactamases produced by the *M. catarrhalis* not only protect the pathogen but also inactivate penicillin, an antibiotic that is commonly used for the treatment of mixed infections caused by other airway pathogens such as *Streptococcus pneumoniae* and/or nontypeable *Haemophilus influenzae* [1, 4].

Molecular investigations reveal the presence of two types of beta-lactamases in *M. catarrhalis*, BRO-1 and BRO-2 that are encoded by two genes *bro-1* and *bro-2*, respectively. These two enzymes can be distinguished by the presence of a 21-base pair (bp) deletion in the promoter region of the *bro-2* gene when comparing the same region in the *bro-1* gene [7, 8].

## 2. Materials and Methods

*M. catarrhalis* was isolated from respiratory clinical samples such as sputum, tracheal aspirates, and bronchoalveolar lavage (BAL) collected from December 2012 to December 2013 in the Microbiology Department, St. John's Medical College, Bangalore. Clinical data for the patients from whom these strains were isolated were collected retrospectively. Ethical clearance for the study was obtained from the Institutional Ethics Committee (IEC) of St. John's Medical College (IEC study Ref no: 201/2018).

### 2.1. Microbiological Methods

**Figure 1:**
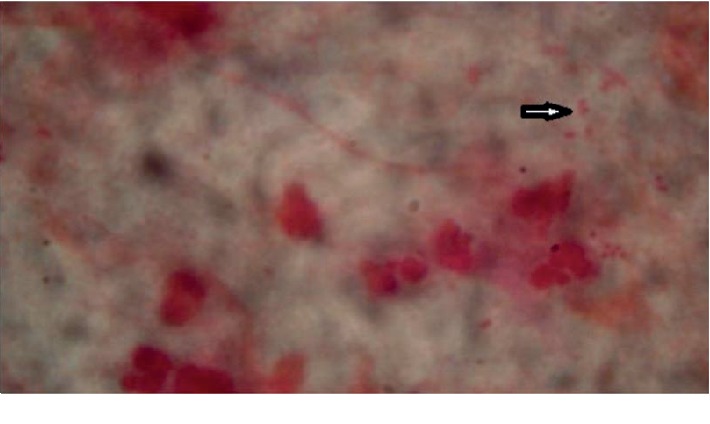
Gram-stained smear showing pus cells and Gram-negative *M. catarrhalis.*

Samples (sputum, tracheal trap, and BAL) were subjected to Gram staining (Figure 1) and then plated on Blood Agar (BA) and Chocolate Agar (CA) with bacitracin disc and MacConkey Agar (MA), following the standard operating procedure of the laboratory. Identification of the isolates was done by following the standard microbiological techniques.

Colony morphology on the BA and CA was nonlytic, round, opaque, convex, and greyish white with no growth observed on MA. The colonies remained intact when pushed across the surface of the agar (hockey puck appearance).

The colonies were confirmed using Gram staining and biochemical tests for catalase, oxidase, DNAse, and butyrate esterase production (Remel™ catarrhalis test disc). This tributyrin spot test helped in differentiating *M. catarrhalis* from other nonpathogenic *Neisseria* species that are generally found in the respiratory tract.

Antibiotic susceptibility testing was done on Mueller Hinton BA using E-strips. Antimicrobial agents used were amoxicillin/clavulanic acid, cefuroxime, cefpodoxime, cefixime, azithromycin, clarithromycin, ciprofloxacin, and levofloxacin. Beta-lactamase production testing was done using the Nitrocefin disc method. Antimicrobial agents used were selected according to the Clinical and Laboratory Standards Institute (CLSI) guidelines.

### 2.2. Molecular Methods

DNA was extracted from the *M. catarrhalis* isolates using the Qiagen Kit. Extraction was performed as per the instructions that were given on the kit. primers used to detect *bro* genes were BROF (5′-TRGTGAAGTGATTTTKRRMTTG-3′) and BROR (5′-GCAATTTATTAACTGGATG TA-3′). The polymerase chain reaction (PCR) protocol followed was as follows: 94°C for 5 min followed by 37 cycles of 94°C for 30 s, 51°C for 90 s, and 72°C for 30 s with a final extension at 72°C for 7 min. The PCR-amplified products were subjected to gel electrophoresis analysis. Gene sequencing was performed on the PCR-amplified products for confirming their identity. The difference in base pairs was studied in the target region for the identification and characterisation of *bro-1* and *bro-2* genes.

## 3. Results

**Figure 2:**
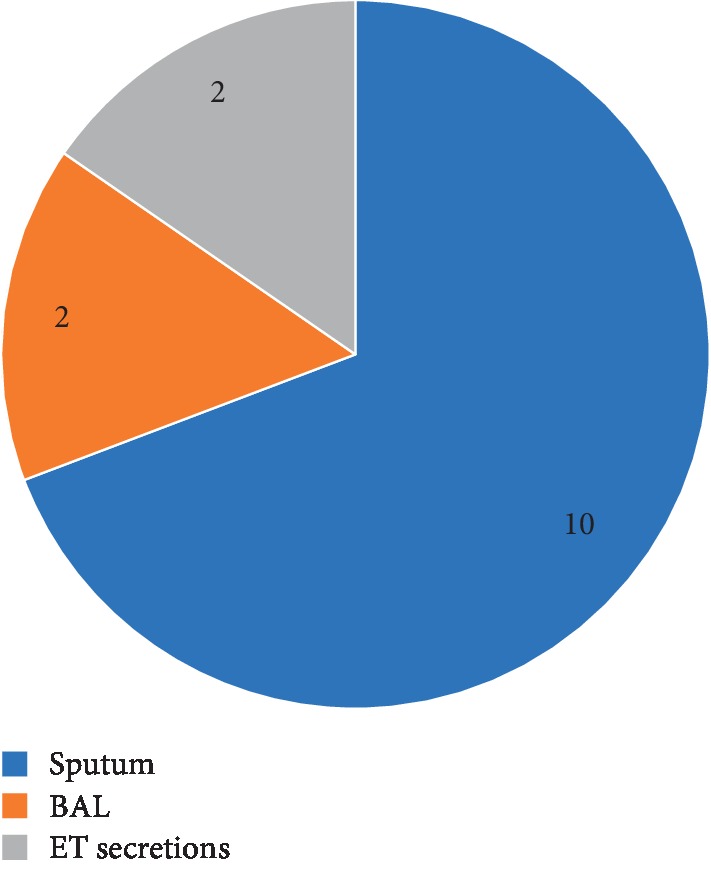
Pie chart showing sample distribution.

We were able to isolate 14 strains of *M. catarrhalis* primarily from sputum samples and less frequently from BAL and tracheal trap samples (Figure 2). These samples on Gram staining showed the presence of pus cells and abundant Gram-negative coccobacilli as seen in Figure 1.

**Figure 3:**
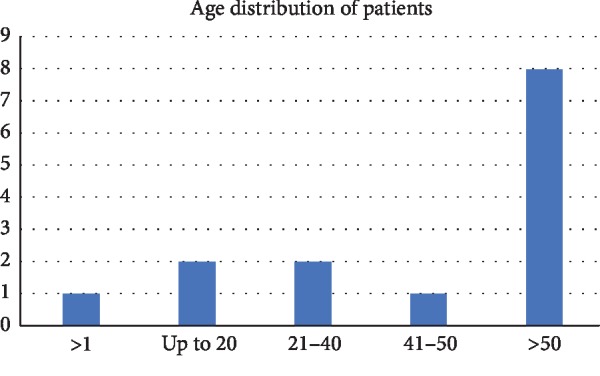
Age distribution of patients in whom *M. catarrhalis* was isolated.

Approximately 72% of the isolates were obtained from male patients. 64% of the isolates were from patients above 40 years of age (Figure 3) and most were associated with chronic obstructive pulmonary disease (COPD), asthma, pneumoniae, or related conditions (Table 1). In approximately 40% of the cases, *M. catarrhalis* was associated with either *S. pneumoniae* or *H. influenzae.*

**Figure 4:**
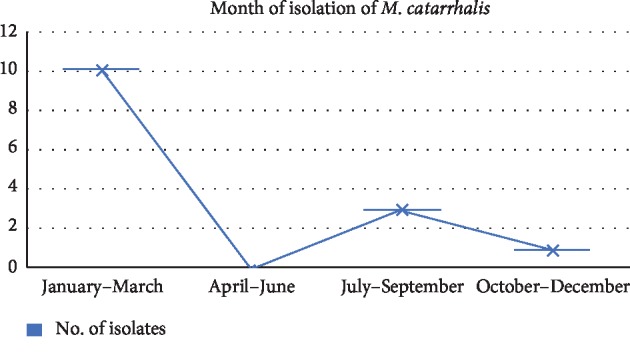
Seasonal distribution of the isolates.

A total of 13 patients with infections with *M. catarrhalis* were managed on an inpatient basis, and one patient was treated on an outpatient basis. Most infections were recorded in the winter season (11 isolates) while 3 infections were reported in the rainy season (Figure 4).

### 3.1. Clinical Characteristics of the Patients from Whom *M. catarrhalis* Was Isolated

All of the fourteen patients included in this study had at least one predisposing clinical conditions that we presume could have led to the secondary infection with the organism. While seven out of the fourteen patients had localised respiratory conditions such as COPD, asthma, and bronchopneumonia, the remaining seven had systemic immunocompromised conditions such as systemic lupus erythematosus (SLE) and acute lymphatic leukaemia (ALL) as enumerated in Table 1.

### 3.2. Antibiotic Susceptibility Pattern and Beta-Lactamase Production

**Figure 5:**
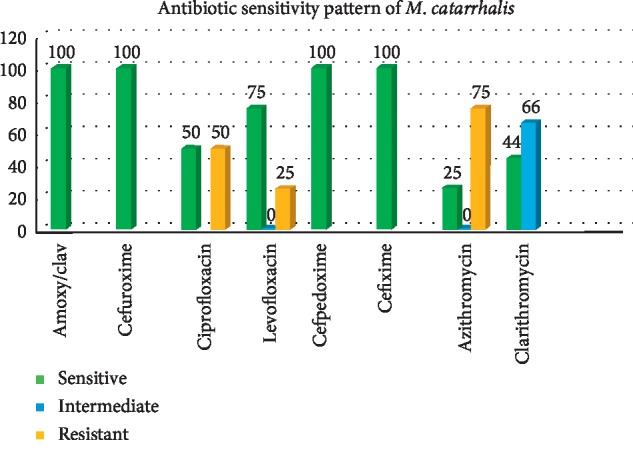
Antibiotic sensitivity pattern of *M. catarrhalis*.

All the fourteen isolates (100%) were identified to be beta-lactamase producers and showed susceptibility to amoxicillin/clavulanic acid combination (Figure 5). Although all the isolates were sensitive to second- and third-generation cephalosporins, the isolates displayed resistance to other groups of drugs. While 25% of the isolates were resistant to levofloxacin, 50% were resistant to ciprofloxacin among drugs belonging to the fluoroquinolone group. 75% of the isolates were resistant to azithromycin, and 66% showed intermediate results with clarithromycin in the macrolide group and all these are determined by the minimum inhibitory concentration (MIC) values using the CLSI guidelines.

**Figure 6:**
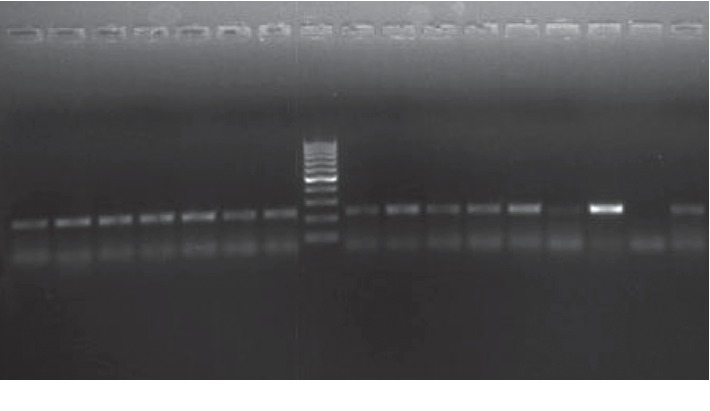
Gel electrophoresis analysis showing the presence of *bro-1*in all isolates.

The samples were subjected to the PCR assay for exploring the presence of *bro-1* and *bro*-*2* genes. All the collected strains showed the presence of *bro-1* beta-lactamase genes. None of the isolates in our study had *bro-2* gene (Figure 6).

## 4. Discussion

*M. catarrhalis* has been considered to be a harmless commensal of the upper respiratory tract for the most part of the 20th century. Only recently, it has gained the status of a pathogen. Many laboratories still do not report *M. catarrhalis* as a pathogen, especially when a better-recognised pathogen such as *S. pneumoniae* or *H. influenzae* is isolated in the same sample containing *M. catarrhalis*. Another issue while considering *M. catarrhalis* as a pathogen is that the colonies of *M. catarrhalis* closely resemble those of *Neisseria* sp., which are normal flora in the respiratory tract. Despite all these factors, in the past decade, the bacterium has emerged as a true pathogen and considered important when isolated [4].

In our study, 72% of the *M. catarrhalis* isolates were from males, and 28% were from females. A similar gender distribution showing a male preponderance has been observed in other studies [9, 10]. Majority of strains isolated in our study were from patients above 40 years of age. In line with our findings, Hager et al. [11], in 1987, summarised in their literature review that the mean age of the patients infected with *M. catarrhalis* was 64.8 years. The higher incidence of *M. catarrhalis* infections in males can be attributed to the decreased systemic and local immunity in the respiratory tract due to smoking and alcohol consumption and predisposing chronic airway diseases that are common in older males. The risk factors other than advanced age, history of smoking, and COPD included immunocompromised patients who were on treatment for autoimmune diseases like SLE and malignancy. Factors such as postsurgical status and postvaricella infections were also observed as risk factors.

Studies by Tamang et al. revealed that the incidence of *M. catarrhalis* infections was found to be the highest in the month of January [10]. This finding is similar to those observed by Felix et al. in 1990 [12], who reported a high incidence of *M. catarrhalis* infections during the winter months. The high incidence of respiratory viral infections in autumn and winter might have a role in weakening the defence system of the respiratory tract, thereby predisposing to infection with low-grade pathogens like *M. catarrhalis* during the winter season. Jakubicz and Leszczyńska [13], in 1997, also found that the frequency of *M. catarrhalis* infections was higher in the autumn-winter period than in summer [10]. In our study, all the isolates were obtained in colder and wetter months.

Antibiotic susceptibility testing conducted in this study revealed that all the *M. catarrhalis* isolates were beta-lactamase producers. Studies from Australia, Europe, and the United States have reported beta-lactamase production in over 90% of the similar isolates [14]. In *M. catarrhalis*, two types of beta-lactamases are found that are phenotypically identical and encoded by the BRO-1 and BRO-2 types of chromosomal genes. Fortunately, both the enzymes are readily inactivated by beta-lactamase inhibitors, and all the isolates were susceptible to amoxicillin in combination with clavulanic acid [4]. Similar observations were recorded in our study. The beta-lactamases from *M. catarrhalis*, when excreted, are known to inactivate penicillin, thereby protecting other associated pathogens as well (in case of mixed infections) [4]. In our study, we too found that *M. catarrhalis* was associated with either *S. pneumoniae* or *H. influenzae* in approximately 40% of the cases, and since all the *M. catarrhalis* isolates were beta-lactamase producers, the enzyme could have inactivated the penicillin used for the treatment of these obligatory respiratory pathogens in these patients.

Although all the strains isolated in this study were uniformly susceptible to cefuroxime, cefpodoxime, and cefixime, 25% of the strains were resistant to levofloxacin and 50% were resistant to ciprofloxacin. Also, azithromycin resistance was shown by 75% of the isolates, and intermediate resistance to clarithromycin was shown by 66% of the isolates. The strains in the present study showed higher resistance to antibiotics when compared with that observed in other studies [14–16]. The resistance to macrolides and quinolones observed in our study may be attributed to the increased use of these oral antibiotics for empirical treatment of common upper respiratory tract infections and lower respiratory tract infections in the community. The availability of “over-the-counter” antibiotics and their nonprescription use by the public could have also contributed to the development of this resistance pattern. This trend, if left unchecked, has the potential to pave the way for the rapid emergence of multiple drug-resistant strains with serious implications.

In the current analysis, we could detect BRO-1 beta-lactamase genes from all isolates, and BRO-2 was not found in any isolate. Presence of *bro*-1 gene is associated with a higher MIC value when compared with *bro*-2 that is attributed to the genetic variation of a 21 bp deletion in the promoter region of BRO-2 [7, 8]. Studies by Mohager et al. have also found the presence of only BRO-1 beta-lactamases in their isolates [17]. However, the presence of BRO-2 beta-lactamase has been reported in some other studies, though the incidence rate was low [3, 18, 19]. The difference may be due to the geographical distribution of the clonally related strain types. We have not found any study from India that has described the prevalence of these genes. However, a study on a large number of samples and isolates is required to confirm the true prevalence of the *bro* genes and their implications in this region.

Taking into the consideration that *M. catarrhalis* has evolved as a beta-lactamase producer and has shown increased resistance to a number of antibiotics, importance should be given whenever *M. catarrhalis* is isolated in respiratory samples. Constant vigilance on the emerging resistance pattern in different microbial species should be maintained in healthcare settings. The resistance pattern and beta-lactamase production of *M. catarrhalis* should be reported along with other primary airway pathogens, whenever coisolated, to decide on the appropriate treatment for symptomatic patients, especially those with other predisposing factors described earlier.

## 5. Conclusions

*M. catarrhalis* was earlier considered a nonpathogenic member of the resident flora of the nasopharynx. Over the past decade, the bacterium has emerged as a genuine pathogen, especially in patients with obstructive airway diseases. *M. catarrhalis* has also been reported to cause upper respiratory tract infections in otherwise healthy children and elderly individuals.

This study shows that *M. catarrhalis* was most commonly isolated from patients in the age group of 40–60 years, especially when associated with predisposing conditions like COPD. The increase in antibiotic resistance and beta-lactamase production in *M. catarrhalis* is a cause of concern. Other options in therapy like azithromycin and clarithromycin may not be effective in treatment due to the increasing resistance as seen in this study. All of the isolates are seen to have gene encoding for beta-lactamase, and the worldwide prevalence of *bro* gene carrying isolate is being increasingly reported. The emerging resistance pattern emphasises the need for antibiotic surveillance and appropriate antibiotic stewardship program to salvage the currently available antibiotics and a new class of drugs to treat serious infections with this organism.

## Figures and Tables

**Table 1:** Clinical condition of patients from whom *M. catarrhalis* was isolated.

S. no.	Clinical condition	Number of patients
1	COPD	2
2	Bronchopneumonia	2
3	Asthma	1
4	COPD with the H/O smoking	1
5	Pulmonary TB with cavities	1
6	SLE	2
7	ALL	1
8	Postoperative status	1
9	Others	3

^*∗*^COPD: chronic obstructive pulmonary disease; SLE: systemic lupus erythematosus; ALL: acute lymphatic leukaemia.

## Data Availability

The data used to support the findings of this study are included within the article.
